# Analysis of the impact of sex and age on the variation in the prevalence of antinuclear autoantibodies in Polish population: a nationwide observational, cross-sectional study

**DOI:** 10.1007/s00296-021-05033-9

**Published:** 2021-11-10

**Authors:** Paweł Krzemień, Sławomir Kasperczyk, Maciej Banach, Aleksandra Kasperczyk, Michał Dobrakowski, Tomasz Tomasik, Adam Windak, Mirosław Mastej, Alberico Catapano, Kausik K. Ray, Dimitri P. Mikhailidis, Peter P. Toth, George Howard, Gregory Y. H. Lip, Maciej Tomaszewski, Fadi J. Charchar, Naveed Sattar, Bryan Williams, Thomas M. MacDonald, Peter E. Penson, Jacek J. Jóźwiak, LIPIDOGRAM2015 Investigators, B. Al-Shaer, W. Andrusewicz, M. Andrzejczuk-Rosa, E. Anusz-Gaszewska, A. Bagińska, P. Balawajder, G. Bańka, E. Barańska-Skubisz, B. Barbara Przyczyna, S. Bartkowiak, J. Bartodziej, M. Bartosiewicz, M. Basałyga, A. Batyra, A. Bąk, M. Bednarz, K. Bejnar, W. Bernacki, M. Betiuk-Kwiatkowska, S. Biegaj, M. Bień, W. Bilski, M. Biłogan, G. Biruta-Pawłowska, A. Biskup, B. Błaszczyk, H. Błaszczyk, T. Błońska-Jankowska, B. Bogacka-Gancarczyk, M. Bojanowska, E. Bonda, J. Borowik-Skwarek, J. Borowska, J. Bruckner, J. Brzostek, M. Brzuchacz, M. Budzyńska, I. Bulzacka-Fugiel, J. Bulzak, K. Bunikowski, A. Cebulska, T. Celka, E. Cempel-Nowak, W. Chechliński, A. Chludzińska, D. Chmiel, M. Chmielewska, M. Cichy, A. Ciemięga, A. Ciepluch, I. Cieszyńska, B. Czajka, B. Czapla, M. Czerner, B. Czerwińska, W. Czuryszkiewicz, E. Daleka, Z. Dawid, M. Dąbrowska, R. Dąbrowska, D. Dąbrowski, M. Dąbrowski, K. Demczyszyn, A. Dębowska-Serwińska, J. Dmochowski, J. Dobrzecka-Kiwior, E. Dolanowska, H. Dolanowski, P. Dołek, M. Domagała, H. Domański, A. Doszel, D. Duda, M. Dudkowska, B. Dudziuk, P. Dybciak, M. Dymanowski, L. Dziadzio-Bolek, M. Eicke, H. El-Hassan, A. Eremus, M. Fąferek-Muller, E. Figura-Roguska, I. Fijałkowska-Kaczmarek, M. Flis, T. Florczak, M. Florczuk, E. Foryszewska-Witan, W. Frydrych, A. Fugiel, E. Futyma, A. Gaca-Jaroszewicz, I. Gajdamowicz, K. Ganczarski, A. Gatnar, M. Gers, A. Głowacki, K. Głód, J. Godula, J. Gołąb, M. Gołębiewski, E. Goszczyńska, K. Gościcka, A. Górna-Hajduga, E. Górny, T. Grabowska, R. Grabowski, A. Graczyk-Duda, A. Gromow, A. Grudewicz, J. Gruszecka, A. Gruszka, J. Gryboś, J. Grzebyk, A. Grzechowiak, D. Grzesiak, T. Grześkowiak, A. Guźla, G. Hachuła, B. Hawel, H. Hiltawska, E. Honkowicz, J. Ignatowicz, K. Imielski, A. Iwaniura, A. Jagieła-Szymala, M. Jalć-Sowała, A. Janczylik, E. Janisz, M. Janiszek, K. Jankiewicz-Ziobro, K. Januszewska, A. Jaremek, A. Jaros-Urbaniak, J. Jarosz, P. Jarosz, W. Jasiński, M. Jezierska-Wasilewska, T. Jędraszewski, A. Jędrzejowska, R. Józefowicz, K. Juźwin, E. Kacprzak, J. Kaczmarek-Szewczyk, M. Kaczmarzyk, R. Kandziora, C. Kaniewski, L. Karolak-Brandt, S. Kasperczyk, E. Kasperek-Dyląg, I. Kedziora, A. Kępa, J. Kiciński, J. Kielak-Al-Hosam, Ł. Kiełczawa, P. Kilimowicz, K. Kitliński, T. Kiwka, U. Klein, L. Klichowicz, A. Klimowicz, B. Klonowski, B. Kmolek, E. Kobyłko-Klepacka, A. Kocoń, A. Kolenda, E. Kollek, M. Kopeć, B. Koper-Kozikowska, J. Koralewska, M. Korczyńska, M. T. Korzeniewski, A. Kosk, K. Kotarski, E. Kowalczyk, M. Kowalczyk, I. Kowalik, B. Kozak-Błażkiewicz, M. Kozik, D. Kozłowska, E. Kozłowska, M. Kozłowska, T. Kozubski, K. Kózka, L. Kraśnik, T. Krężel, B. Krochmal, B. Król, G. Król, J. Król, T. Królikowska, H. Kruszewska, B. Krygier-Potrykus, W. Krystek, J. Krzysztoń, T. Kubicki, A. Kuczmierczyk-El-Hassan, W. Kuczyńska-Witek, D. Kujda, A. Kurowski, I. Kurzelewska-Solarz, M. Kwaczyńska, M. Kwaśniak, P. Kwaśniak, T. Kwietniewska, A. Łebek-Ordon, A. Lebiedowicz, L. Lejkowska-Olszewska, M. Lentas, A. Lesiewicz-Ksycińska, M. Limanowski, S. Łoniewski, J. A. Łopata, B. Łubianka, I. Łukasiuk, M. Łużna, M. Łysiak, B. Łysik, Z. Machowski, J. Maciaczyk-Kubiak, G. Mackiewicz-Zabochnicka, Z. Magner-Krężel, S. Majda, P. Malinowski, J. Mantyka, E. Marchlik, G. Martyna-Ordyniec, J. Marzec, M. Marzec, R. Matejko-Wałkiewicz, M. Mazur, M. Michalczak, A. Michalska-Żyłka, M. Michniewicz, D. Mika-Staniszewska, E. Mikiciuk, T. Mikołajczak, J. Milewski, E. Miller, B. Misiaszek, M. Mizik-Łukowska, E. Młyńczyk-Pokutycka, M. Mocek, M. Moczała, M. Morawska-Hermanowicz, P. Moryc, A. Moskal, S. Moskal, A. Moździerz, P. Moździerz, M. Mrozińska, K. Mrozowicz, G. Mróz, T. Munia, A. Mura, M. Muras-Skudlarska, E. Z. Murawska, Ł. Murawski, R. Murawski, R. Musielak, K. Nadaj, W. Nagarnowicz, R. Napierała, M. Niedźwiecka, A. Niemirski, J. Nikiel, M. Nosal, W. Nowacki, J. Nowak, M. Nyrka, A. Obst, J. Ochowicz, E. Ogonowska, M. Oleszczyk, A. Ołdakowski, I. Ołowniuk-Stefaniak, J. Ordowska-Rejman, M. Orliński, B. Osińska, A. Ostańska-Burian, A. Paciorkowska, U. Paczkowska, L. Paluch, L. Pałka, J. Paszko-Wojtkowska, A. Paszkowska, E. Pawlak-Ganczarska, W. Pawlik, I. Pawłowska, M. Paździora, G. Permiakow, A. Petlic-Marendziak, T. Piasecka, E. Piaścińska, A. Piktel, A. Pilarska-Igielska, A. Piotrkowska, K. Piwowar-Klag, M. Planer, J. Plewa, P. Płatkiewicz, B. Płonczyńska, A. Podgórska, M. Polewska, B. Porębska, P. Porwoł, I. Potakowska, A. Prokop, J. Przybylski, M. Przybyła, H. Psiuk, K. Ptak, G. Puzoń, N. Rabiza, S. Rachwalik, E. Raczyńska, M. Raniszewska, A. Romanek-Kozik, A. Rosa, K. Rosa, A. Rozewicz, J. Rudzka-Kałwak, J. Rusak, D. Rutkowska, M. Rybacki, D. Rybińska, A. Rycyk-Sadowska, L. Rynda, B. Rynkiewicz, B. Sadowska-Krawczyk, M. Sadowska-Zarzycka, B. Sarnecka, E. Sawalach-Tomanik, B. Sidor-Drozd, M. Siemieniak-Dębska, A. Sieroń, B. Siewniak-Zalewska, A. Sikora, B. Sitarska-Pawlina, J. Skorupski, I. Skrzypińska-Mansfeld, J. Skubisz, R. Skwarek, M. Słodyczka, M. Smentek, K. Smolińska, B. Solarz, W. Sosnowska, B. Sroka, H. Stachura, D. Stangreciak, M. Staniak, Z. Stańczyk, D. Stańszczak-Ozga, E. Startek, M. Stefańczyk, R. Stelmach, E. Sternadel-Rączka, M. Sternik, J. Stępień, J. Stocka, M. Stokowska-Wojda, M. Studler-Karpińska, W. Suchorukow, W. Sufryd, B. Supłacz, J. Sygacz, Ł. Szczepański, J. Szkandera, J. Szłapa-Zellner, D. Szydlarska, T. Śliwa, J. Śliwka, Ł. Śmiejkowski, A. Targońska, E. Tesarska, M. Tobiasz, J. Tomaka, K. Tomalska-Bywalec, E. Tomiak, S. Topczewski, A. Trawińska, L. Trela-Mucha, D. Trojanowski, M. Trzaskowska, B. Trzcińska-Larska, A. Trznadel-Mozul, K. Ulanicka-Liwoch, M. Urbanowicz, A. Uthke-Kluzek, J. Waczyński, J. Walczak, L. Warsz, M. Wasyńczuk, U. Wąchała-Jędras, D. Wąsowicz, J. Wczysła, F. Wenda, E. Werner-Kubicka, E. Weryszko, B. Węgrzynowska, M. Wiaksa, M. Wiankowski, A. Wicherek, R. Wieczorek, R. Wiencek, G. Wienzek-Tatara, B. Wierzbicka, M. Wierzbicki, B. Wilczyńska, D. Wilmańska, P. Winiarski, A. Wiszniewska-Pabiszczak, M. B. Witkowska, J. Witzling, A. Wlaź, I. Wojtkowiak, J. Woydyłło, K. Woźniak, A. Wójtowicz, J. Wrona, M. Wrońska, H. Wujkowska, J. Wyrąbek, O. Wysokiński, R. Zakrzewski, J. Zaleska-Zatkalik, J. Zaleski, M. Zalewska-Dybciak, E. Zalewska, B. Zalewska-Uchimiak, J. Zawadzka-Krajewska, J. Zawadzki, A. Zieliński, E. Zubrycka, I. Żybort, M. Żymełka

**Affiliations:** 1 Euroimmun Polska Sp. z o.o., 2a Widna St., 50-543 Wrocław, Poland; 2 Department of Biochemistry, Faculty of Medical Sciences in Zabrze, Medical University of Silesia, Katowice, Poland; 3 Department of Hypertension, Medical University of Lodz, Łódź, Poland; 4 Department of Family Medicine, Jagiellonian University Medical College, Kraków, Poland; 5 Mastej Medical Center, Jasło, Poland; 6 Department of Pharmacological Sciences, University of Milano and Multimedica IRCCS, Milan, Italy; 7 Imperial Centre for Cardiovascular Disease Prevention, Department of Primary Care and Public Health, Imperial College, Kensington, London, UK; 8 Department of Clinical Biochemistry, Royal Free Hospital, University College London, London, UK; 9 Cicarrone Center for the Prevention of Cardiovascular Disease, Johns Hopkins University School of Medicine, Baltimore, MD USA; 10 CGH Medical Center, Sterling, IL USA; 11 Department of Biostatistics, School of Public Health of Alabama, Birmingham, AL USA; 12 Liverpool Centre for Cardiovascular Science, University of Liverpool and Liverpool Heart and Chest Hospital, Liverpool, UK; 13 Department of Clinical Medicine, Aalborg University, Aalborg, Denmark; 14 Division of Cardiovascular Sciences, Faculty of Biology, Medicine and Health, University of Manchester, Manchester, UK; 15 School of Health and Life Sciences, Federation University Australia, Ballarat, VIC Australia; 16 Institute of Cardiovascular and Medical Science, University of Glasgow, Glasgow, UK; 17 NIHR University College London Biomedical Research Centre, University College London and University College London Hospitals NHS Foundation Trust, London, UK; 18 MEMO Research, University of Dundee, Ninewells Hospital and Medical School, Dundee, DD1 9SY UK; 19 School of Pharmacy and Biomolecular Sciences, Liverpool John Moores University, Liverpool, UK; 20 Liverpool Centre for Cardiovascular Science, Liverpool, UK; 21 Department of Family Medicine and Public Health, Faculty of Medicine, University of Opole, Opole, Poland

**Keywords:** Antinuclear antibodies, Prevalence, Indirect immunofluorescence assay, Sex, Age, Cutoff threshold

## Abstract

The detection of antinuclear autoantibody (ANA) is dependent on many factors and varies between the populations. The aim of the study was first to assess the prevalence of ANA in the Polish adult population depending on age, sex and the cutoff threshold used for the results obtained. Second, we estimated the occurrence of individual types of ANA-staining patterns. We tested 1731 patient samples using commercially available IIFA using two cutoff thresholds of 1:100 and 1:160. We found ANA in 260 participants (15.0%), but the percentage of positive results strongly depended on the cutoff level. For a cutoff threshold 1:100, the positive population was 19.5% and for the 1:160 cutoff threshold, it was 11.7%. The most prevalent ANA-staining pattern was AC-2 Dense Fine speckled (50%), followed by AC-21 Reticular/AMA (14.38%) ANA more common in women (72%); 64% of ANA-positive patients were over 50 years of age. ANA prevalence in the Polish population is at a level observed in other highly developed countries and is more prevalent in women and elderly individuals. To reduce the number of positive results released, we suggest that Polish laboratories should set 1:160 as the cutoff threshold.

## Introduction

Autoimmune diseases (AD) are characterized by immune responses to self-antigens resulting in tissue damage or dysfunction. The response can be systemic or can affect specific organs or body systems [1]. AD include more than 80 diseases, of which systemic autoimmune rheumatic diseases (SARDs) are a particular group, with a very diverse clinical picture and complex pathogenetic mechanisms [1]. It is well known that SARDs mainly affect older people, and predominantly women [2–5]. At the same time, there are increasing cases of connective tissue diseases in young people and even in children [6–8]. Thus far, the etiology of antinuclear antibody (ANA) formation has not been fully elucidated. There are many suspected factors that increase the risk of ANA biogenesis and occurrence, including genetic predisposition and environmental factors such as infections, oxidative stress factors, physical and chemical agents, as well as stressful life events [4, 5, 9–11].

Laboratory diagnosis of SARDs is complicated by the requirement for specialized tests to enable detection of autoantibodies. For years, the indirect immunofluorescence assay (IIFA) has been regarded as the gold standard for measuring ANA. According to current recommendations, the human laryngeal carcinoma cell line (HEp-2) is a recommended substrate for detecting ANA, and each positive test result should include an estimated titer and fluorescence pattern [6]. The current recommendation for a positive ANA finding is a titer of ≥ 1:160 [6]. However, the IIFA assay ANA threshold titer is dependent on reagents, equipment and other local factors, and hence the screening dilution should be defined locally.

A main feature of the screening test should be high sensitivity (which significantly reduces its specificity, i.e., the number of false-positive results increases—positive results are also obtained in people without symptoms of rheumatic disease) [12]. This means that the higher the antibody titer, the greater the likelihood that the result is clinically relevant and is associated with symptoms.

Autoimmunity is not always clinically symptomatic and can be observed in individuals without clinical manifestations of disease. In this case, so-called natural autoantibodies (NAAbs) can be observed in the patient’s serum, which bind with low avidity epitopes naturally existing in the patient’s body [13, 14]. In genetically predisposed individuals, chronic activation of the immune system can lead to proliferation of autopolymerizing B-lymphocyte clones, increased autoantibody titres and finally to the development of clinical symptoms of autoimmune disease [15, 16]. The prevalence of autoantibodies in the general population has been estimated to be in the range 5.92–30.8%, but this wide range is mainly due to differences in the cutoff values used in the studies and the population studied, e.g., autoantibodies are more common in African-Americans and less common in the Chinese population. [2, 4, 17–22].

The aim of this study was first to assess the prevalence of ANA in the Polish population, taking into account age, sex and the cutoff threshold used for the results obtained. Second, we estimated the occurrence of individual types of ANA-staining patterns. As far as we are aware, similar large cohort population studies have not yet been conducted in the Polish population.

## Materials and methods

### Design

A nationwide observational, cross-sectional study was carried out in Poland in the fourth quarter of 2015 and the first and second quarters of 2016.

### Sampling

This study is part of a large research program “Nationwide study of cardiovascular health in primary care in Poland—LIPIDOGRAM2015 and LIPIDOGEN2015”, the design and rationale of which have been described in detail previously [23]. Briefly, the recruitment was carried out by 438 physicians-investigators in 398 primary care practices recruited in 16 (of 17) voivodeships (major administrative region in Poland), in line with the operational structure created by previous studies [24, 25]. Physicians-investigators were randomly selected from the Medical Data Management database. Each physician-investigator selected at least 30 patients in each Primary Health Care practice for participation in the LIPIDOGRAM2015 study. From each group of 30 patients, up to 4 patients were randomly selected by the physician-investigator to participate in the LIPIDOGEN2015 sub-study. The expected number of patients recruited for LIPIDOGRAM2015 study (consecutive sample) was 13,000–14,000 with 13–15% (1700–2000) enrolled to the LIPIDOGEN2015 sub-study (random sample). The program covered only adult patients over 18 years old. For each patient recruited for the study, a 28-item questionnaire was collected containing data on chronic diseases and their treatment, lifestyle (diet, physical activity, smoking) and family history of cardiovascular diseases (CVDs) (24 questions in total). The questionnaire also included demographic data: age, gender, place of residence and level of education (4 questions in total). To avoid missing data, only the most important data elements were collected to minimize the burden and focus on routinely collected data. The questionnaire was tested with a group of 10 primary care physicians who had no comments or difficulties in completing it. Content validity was checked by comparing the questionnaire with other similar tools used in Poland (an English translation of the questionnaire can be found in the LIPIDOGRAM2015 study design) [23]. Each questionnaire was labeled with an individual barcode, identical to the barcodes on samples of blood and saliva. Anthropometric measurements (height, body weight, waist circumference, and hip circumference) were performed at the doctor’s office. In all enrolled patients, serum samples were obtained after ≥ 12 h of fasting by collecting whole blood from an antecubital vein. On the same day, measurements of blood pressure, heart rate, and fasting glucose were obtained as well as lipid profile samples. For the LIPIDOGEN2015 sub-study, saliva samples for DNA isolation and blood samples for measurement of glycated hemoglobin, oxidative stress parameters, autoantibody levels, and inflammatory cytokine profile and apolipoprotein profile were collected.

For this study, we used 1731 serum samples from the abovementioned LIPIDOGEN2015 sub-study. The tested group included 1043 women and 688 men. The blood samples were transferred in cooled containers (−20 °C) to a central laboratory (Silesian Analytical Laboratories—SLA in Katowice, Poland) for biochemical analyses and then to the autoimmune laboratory (Euroimmun Poland Ltd. Customer Training Laboratory in Wroclaw, Poland) for ANA determination.

### Laboratory analyses of ANA

ANAs were detected by a IIFA using human laryngeal carcinoma cells (HEp-2) with commercially available Euroimmun Medizinische Labordiagnostika AG (Lübeck, Germany) test kits Mosaic Basic Profile 3 (catalogue number FC 1800-2010-3). Sample incubation was carried out manually, according to the instructions provided by the manufacturer of the test, except that 998 samples were diluted with a threshold cutoff 1:160 as recommended by the current guidelines [6] and 733 patient samples were diluted with a threshold cutoff 1:100 as recommended by the manufacturer’s instruction. The samples were randomly divided into two groups. The results were evaluated using a EUROstar III fluorescence microscope (CarlZeiss Oberkochen, Germany). The test results were evaluated by an experienced technician. The test result included a qualitative assessment of the presence of ANA, estimation of antibody titer, and determination of the characteristic pattern according to the International Consensus on ANA patterns (ICAP) nomenclature [26]. The results from IIFA were collected and stored as digital images.

### Statistical analysis

Statistical analyses was carried out were performed using Statistica 13.3 (StatSoft, Tulsa, USA). Data are expressed as mean ± SD (for normal distribution) and median (nonparametric distribution) for continuous variables, and as a percentage for categorical variables. Univariate comparison of markers related to autoimmune diseases according to clinical variables was performed using the *U*-Mann–Whitney method for nonparametric variables or χ^2^ test/Fisher exact test where appropriate. A two-sided *p* < 0.05 was considered to indicate significance.

## Results

The study included 1731 patients attending primary health care practices (1043 women and 688 men). 1098 people were diagnosed with hypertension, coronary artery disease, dyslipidemia, diabetes, atrial fibrillation, kidney disease or stroke. 649 people were apparently healthy individuals. The mean age of participants was 51 ± (SD 13 years) and 60.25% were female (Table 1). The body mass index (BMI) indicated that the participants were on average slightly overweight [27], and the average waist–hip ratio (WHR) was above the normal range for both men and women [28].

**Table 1:** Characteristics of the population

	All *n* = 1731		Male *n* = 688		Female *n* = 1043	
	Mean	SD	Mean	SD	Mean	SD
Age	51.0	13.0	50.4	13.1	51.6	12.9
Height (cm)	168	9.15	177	6.71	163	6.05
Weight (kg)	80.2	17.1	91.2	15.2	73.0	14.1
BMI (kg/cm^2^)	28.2	5.05	29.3	4.50	27.5	5.29
Waist circumference (cm)	94	14.3	101	12.0	89.3	13.6
Hip circumference (cm)	105	10.8	105	9.33	105	11.7
WHR	0.89	0.09	0.96	0.07	0.85	0.07

*WHR* waist–hip ratio; *BMI* body mass index

**Fig. 1:**
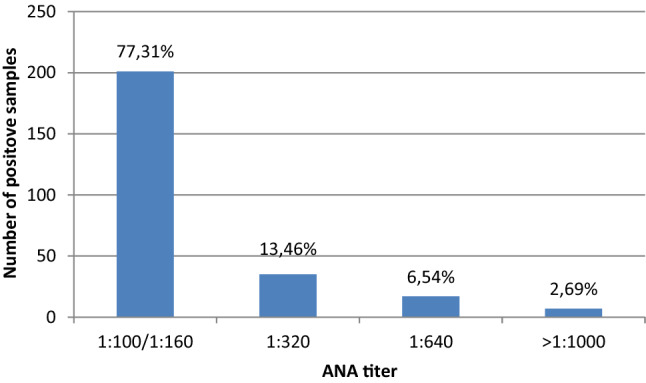
Frequency analysis comparing the 1:100 and 1:160 groups (*n* = 260) in terms of final ANA titer

The ANA test was positive in 260 patients (15.0%) of the entire study population. Of the 733 participants for whom a cutoff threshold of 1:100 was used, 19.5% (*n* = 143) had a positive result for ANA. Only 27 patients in this group had titers higher than 1:100. In the second group, consisting of 998 participants with a 1:160 cutoff threshold, the percentage of ANA-positive results was clearly lower, at 11.7% (*n* = 117). 32 patients in this group had titers higher than 1:160. Frequency analysis comparing the 1:100 and 1:160 groups in terms of final ANA titer is shown in Fig. 1. ANA-staining patterns corresponding to ICAP are in Table 2. We present summary data for both subgroups because the cutoff titer used did not significantly affect the distribution of detected types of staining. The most frequent ANA-staining pattern was AC-2 Dense Fine speckled (50%) followed by AC-21 Reticular/AMA (15.4%) and AC4/AC5—Fine/Large/Coarse Speckled (14.6%).

**Table 2:** Frequency distribution of ANA patterns

ICAP pattern	Number *n* = 260	%
AC-1—homogenous	10	3.85%
AC-2—dense fine speckled	130	50.00%
AC-3—centromere	3	1.15%
AC4/AC5—fine/large/coarse speckled	38	14.62%
AC6/AC7—multiple/few nuclear dots	3	1.15%
AC-8—homogenous nucleolar	1	0.38%
AC-9/AC-10—clumpy/punctate nucleolar	28	10.77%
AC11/AC12—smooth/punctate nuclear envelope	2	0.77%
AC-15—fibrillar linear	6	2.31%
AC-16—fibrillar filamentous	4	1.54%
AC-19/AC-20—dense fine/fine speckled	6	2.31%
AC-21—reticular/AMA	40	15.38%
AC-23—rods and rings	3	1.15%
AC-25/AC-26—spindle fibers/NuMA-like	4	1.54%
AC-27—intercellular bridge	2	0.77%
AC-28—mitotic chromosomal	1	0.38%

*AC* anti-cell; *AMA* antimitochondrial antibodies; *ICAP* International Consensus on ANA patterns; *NuMA* nuclear mitotic apparatus

The relationship between sex and the occurrence of autoantibodies is shown in Table 3. In the tested group, women had an 84% higher risk of a titer of 1:100 than men, and at higher titres the risk was even higher, ranging from 2-fold (for a titer of 1:160) to 3.3-fold (for a titer of 1:640). In general, there were no significant differences in the types of patterns detected in either sex, but with a few notable exceptions. AC4/AC5 antibodies were more than four times as common in women (OR = 4.46) and AC-2 was more than twice as common (OR = 2.33). In contrast, AC-9/AC-10 were more than twice as common in men (OR = 0.42).

**Table 3:** Titers and types of autoantibody staining stratified on gender

	Male		Female		*P* value	OR	− 95% CI	+ 95% CI
	*n* = 688		*n* = 1043					
	Percentage	Number	Percentage	Number				
ANA titer 1:100	10.6%	73	17.9%	187	** < 0.001**	**1.84**	1.38	2.46
ANA titer 1:160	5.4%	37	10.3%	107	** < 0.001**	**2.01**	1.37	2.96
ANA titer 1:320	1.7%	12	4.5%	47	**0.001**	**2.66**	1.40	5.05
ANA titer 1:640	0.6%	4	1.9%	20	**0.020**	**3.34**	1.14	9.84
ANA titer 1:1000	0.1%	1	0.6%	6	0.168	3.97	0.48	33.21
AC4/AC5 fine/large/coarse speckled	0.7%	5	3.2%	33	**0.001**	**4.46**	1.73	11.51
AC-2 dense fine speckled	4.4%	30	9.6%	100	** < 0.001**	**2.33**	1.53	3.54
AC-9/AC-10 clumpy/punctate nucleolar	2.5%	17	1.1%	11	**0.022**	**0.42**	0.20	0.90
AC-1 homogenous	0.4%	3	0.7%	7	0.528	1.54	0.40	5.99
AC-3 centromere	0.1%	1	0.2%	2	0.820	1.32	0.12	14.61
AC6/AC7 multiple/few nuclear dots	0.1%	1	0.2%	2	0.820	1.32	0.12	14.61
AC11/AC12 smooth/punctate nuclear envelope	0.1%	1	0.1%	1	0.767	0.66	0.04	10.58
AC-15 fibrillar linear	0.6%	4	0.2%	2	0.177	0.33	0.06	1.80
AC-16 fibrillar filamentous	0.1%	1	0.3%	3	0.547	1.98	0.21	19.14
AC-19/AC-20 dense fine/fine speckled	0.4%	3	0.3%	3	0.607	0.66	0.13	3.28
AC-21 reticular/AMA	1.6%	11	2.8%	29	0.109	1.76	0.87	3.55
AC-23 rods and rings	0.1%	1	0.2%	2	0.820	1.32	0.12	14.61
AC-8 homogenous nucleolar	0.1%	1	0.0%	0	0.218	–	–	–
AC-25/AC-26 spindle fibers/NuMA-like	0.0%	0	0.4%	4	0.104	–	–	–
AC-27 intercellular bridge	0.3%	2	0.0%	0	0.082	–	–	–
AC-28 mitotic chromosomal	0.0%	0	0.1%	1	0.417	–	–	–

Statistically significant data are in bold

*AC* anti-cell, *ANA* antinuclear antibody; *AMA* antimitochondrial antibodies; *CI* confidence interval; *ICAP* International Consensus on ANA patterns; *NuMA* nuclear mitotic apparatus; *OR *odds ratio

Characteristics of the participants based on the occurrence of ANA are presented in Table 4 which shows that significant factors for ANA positivity include age and gender. Women have an 81% higher risk of positive ANA than men (OR = 1.81 CI 1.35–2.42). Each 1-year increase in age is associated with a 2% (OR = 1.02 CI = 1.01–1.03) increase in the risk of ANA positivity. No correlation was found between the occurrence of autoantibodies and broader cardiovascular disease and lipid disorders. Autoantibodies were more frequently detected in the elderly (*p* < 0.001)—Table 4, differences for individual age ranges are shown in Table 5. In the entire studied population, the lowest percentage of ANA-positive individuals was observed in those under 30 years of age (7.9%), and the highest proportions of ANA-positive people were in those aged 60–70 (20.6%) and over 70 years (22.4%). 84% of ANA-positive individuals were over 40 (*n* = 219) and 64% were over 50 years of age (*n* = 167).

**Table 4:** Demographic and clinical characteristics and the presence of ANA

	ANA negative		ANA positive		Change%	*P* value	OR	− 95% CI	+ 95% CI
	*n* = 1471		*n* = 260						
	Mean/%	SD/*n*	Mean/%	SD/*n*					
Genders (% of men)	42%	615	28%	73		** < 0.001**	**1.84**	1,38	2,46
Age (years)	51	13	54	12	6%	** < 0.001**	**1.02**	1,01	1,03
Height (cm)	169	9.2	166	8.3	−1%	** < 0.001**	**0.97**	0,95	0,98
Weight (kg)	80.7	17.2	77.6	16.2	−4%	**0.008**	**0.99**	0,98	1,00
BMI (kg/m^2^)	28.2	5.05	28.0	5.11	−1%	0.513	0.99	0,97	1,02
Waist circumference (cm)	94.1	14.4	93.0	13.6	−1%	0.257	0.99	0,99	1,00
Hip circumference (cm)	105.0	10.8	105.6	11.1	1%	0.381	1.01	0,99	1,02
WHR	0.90	0.09	0.88	0.08	−2%	**0.013**	**0.16**	0,04	0,68
Chronic kidney disease	2.7%	40	1.5%	4		0.265	0.56	0,20	1,58
Coronary artery disease	10.3%	152	11.2%	29		0.690	1.09	0,71	1,66
Myocardial infarction	4.1%	61	4.2%	11		0.950	1.02	0,53	1,97
Ischemic stroke	1.5%	22	1.9%	5		0.608	1.29	0,48	3,44
Hemorrhagic stroke	0.3%	4	0.4%	1		0.755	1.42	0,16	12,73
Atrial fibrillation	2.8%	41	4.2%	11		0.209	1.54	0,78	3,04
Dyslipidemia	49.4%	726	51.9%	135		0.445	1.11	0,85	1,44
Family hypercholesterolemia	3.7%	55	2.3%	6		0.249	0.61	0,26	1,43
Diabetes mellitus	15.6%	229	19.2%	50		0.139	1.29	0,92	1,81
Arterial hypertension	42.1%	619	47.7%	124		0.092	1.25	0,96	1,64
Healthy individuals	36.3%	534	38.1%	99		0.584	1.08	0,82	1,42
Multivariable-adjusted logistic regression analysis									
Genders (% of men)						** < 0.001**	**1.81**	1.35	2.42
Age (years)						** < 0.001**	**1.02**	1.01	1.03

Statistically significant data are in bold

*ANA* antinuclear antibodies; *BMI* body mass index; *CI* confidence interval; *OR* odds ratio; *WHR* waist–hip ratio

**Table 5:** Characteristics of the population based on age in the occurrence of ANA (* comparison male vs female dependent on age)

Age (years)	All			Male			Female			*p* value (gender)
	Number of individuals	Number of positive ANA	Percentage positive ANA	Number of individuals	Number of positive ANA	Percentage positive ANA	Number of individuals	Number of positive ANA	Percentage positive ANA	
< 30	127	10	7.9%	52	2	3.8%	75	8	10.7%	0.143
30–40	227	31	13.7%	100	8	8.0%	127	23	18.1%	**0.024**
40–50	380	52	13.7%	151	19	12.6%	229	33	14.4%	0.610
50–60	555	74	13.3%	221	17	7.7%	334	57	17.1%	**0.001**
60–70	344	71	20.6%	129	22	17.1%	215	49	22.8%	0.203
> 70	98	22	22.4%	35	5	14.3%	63	17	27.0%	0.138
*p* value (age)	**0.001**			**0.035**			**0.038**			0.038
All	**1731**	**260**	**15.0%**	**688**	**73**	**10.6%**	**1043**	**187**	**17.9%**	

Statistically significant data are in bold

*ANA* antinuclear antibodies

## Discussion

In our study, the prevalence of ANA in the Polish population was 15%, similar to results observed in other developed countries. However, it is worth noting that the percentage of positive results is strongly dependent on the cutoff threshold used. Therefore, to reduce the number of positive results released by Polish laboratories, we suggest that a serum dilution of 1:160 be used for screening purposes, especially since it is very rare for individuals with lower ANA titers to have clinical symptoms [20, 29]. However, for official recommendations to be made, it would be necessary to conduct additional studies to collect additional data on the diagnostic sensitivity and specificity of this approach in a group of patients diagnosed with SARD. Therefore, this proposal should not be considered as an official recommendation to laboratories.

### Prevalence of ANA in the Polish population and the influence of the cutoff threshold used

The prevalence of ANA in the general population is common, and depending on the cutoff threshold used by investigators, can reach up to 30.8% [22]. The aim of the present study was to determine the prevalence of ANA in Polish population based upon on the cutoff threshold used and the influence of patient sex and age on the results. A total of 1731 samples were tested, and ANA were detected in 15%. This result does not differ from the those obtained by other researchers using a similar level of cutoff thresholds, e.g., in the previously mentioned studies of Maritz et al*.* ANA were present in 12.9% of healthy individuals at a 1:80 cutoff titer [29]. Similar results were obtained by Prüßmann et al*.* who tested over 5000 healthy blood donors among which 17.7% were ANA positive [18] and Agmon-Levin et al*.* who reported 13.3% at the 1:80 cutoff titer [6]. ANA prevalence in the U.S. population aged ≥ 12 years was also similar at 13.8% (titer ≥ 1:80) [19]. A higher percentage of ANA was observed by Akmatov et al*.* in Germany, in which the general population was found to be positive for ANA in 33.3% of individuals (titer ≥ 1:80) and 28.6% with a titer of 1:80 or 1:160 [4].

The influence of the cutoff threshold used on the percentage of positive results is obvious. Therefore, our study assessed the impact of the initial cutoff dilution used in the Polish population on the number of positive results obtained. To assess the impact, the test group was divided into two subgroups. In the first group we used the cutoff threshold 1:100 recommended by the test manufacturer. At this cutoff in the examined group of 733 samples, a positive result was observed in 19.5%. A 1:160 cutoff threshold, which is often found to be the most suitable for the evaluation of adult patients [6] was used in the second subgroup where the percentage of ANA-positive results was lower, at 11.7%. This downward has been observed in previous studies [4].

Low antibody titers are usually not clinically relevant and are rarely accompanied by clinical symptoms. In contrast, there is an increased likelihood of SARDs with higher ANA titer [4, 8]. Indeed, 13.5% of our participants had a titer of 1:320, 6.45% titer 1:640 and 2.69% ≥ 1:1000, and these individuals may be at higher risk of developing or suffering from SARDs.

### Prevalence of different types of pattern staining

As reported by Satoh et al*.* in ANA-positive individuals, nuclear patterns were seen in 84.6%, cytoplasmic patterns in 21.8%, and nucleolar patterns in 6.1% [19]. Our results appear to show that according to the ICAP classification, nuclear staining was observed in 77%, cytoplasmic patterns in 20.6% and mitotic in 2.5%. The most frequent ANA-staining pattern was AC-2 dense fine speckled (50%) followed by AC-21 Reticular/AMA (15.38%) and AC4/AC5—Fine/Large/Coarse Speckled (14.62%). The dense fine speckled pattern (AC-2) is associated with apparently healthy individuals, but this association only holds if the specificity is confirmed as monospecific for DFS70 because the pattern recognized as AC-2 is not always induced by anti-DFS70 antibodies [30]. Miyara et al*.* showed also that the likelihood of anti-DFS70 antibodies is significantly lower than in patients with other IIFA patterns [31]. Due to the fact that the AC-2 pattern was detected in half of the samples tested, an interesting issue requiring further testing would be an assessment of the prevalence of anti-DFS70 antibodies in the Polish population.

As mentioned, there were no significant differences for the types of pattern staining by gender except AC4/AC5 and AC-2 which were detected much more often in women than in men. In contrast, nucleolar type of staining AC-9/AC-10 was more often detected in men.

### The presence of ANA in relation to sex and age

It would be useful to link these data with information about the incidence of individual connective tissue diseases. Unfortunately, the survey completed by patients participating in the LIPIDOGRAM2015 and LIPIDOGEN2015 project did not include questions about SARDs. Furthermore, there are no relevant statistical surveys for the Polish population [32]. Among ANA-positive individuals, there was a clear predominance of women over men, consistent with other worldwide observations [3, 5, 19].

On the one hand, it was shown that in the study group the risk of ANA positivity increases with each additional year of life by 2%, but on the other hand, this did not result in an apparent upward trend in the percentage of ANA positivity when comparing the following age groups when divided into 10-year age intervals. In 30- to 60-year-old patients, the percentage of positive results remains at 13.4–13.7% for each 10-year interval. Overall, 84% of ANA-positive patients were over 40 years of age and 64% over 50 years of age. Considering that the risk of ANA positivity in women is up to 81% higher, it is not surprising that in all age ranges, the percentage of ANA positivity in women is higher than in men.

Most people with a positive ANA are not diagnosed with autoimmune disease, and the probability of future disease is low [20] but, as shown by Jonsson et al., sometimes the production of specific autoantibodies (AAb) precedes the symptoms and diagnosis of connective tissue diseases [33].

## Limitations

The present study is limited by a lack of data about the clinical symptoms of SARDs among the participants, so we could not compare the results obtained with any clinical manifestations. To assess the real clinical relevance of the antibodies we detected, details of symptoms experienced by participants would be necessary. Future studies by our research group will also determine the prevalence of anti-DFS70 antibodies in the Polish population and their correlation with gender and clinical symptoms in patients.

*In conclusion*, ANA prevalence in the Polish population is similar to that observed in other highly developed countries. In the Polish population, ANAs are more prevalent in women and with elderly individuals. The cutoff threshold used in the laboratory has a considerable impact on the percentage of positive results obtained. To reduce the number of positive results released, we suggest that Polish laboratories should set 1:160 as the cutoff threshold. However, more studies are needed before this threshold can be incorporated within practice guidelines.

## Data Availability

The datasets generated during and/or analyzed during the current study are available from the corresponding author on reasonable request.

## Abbreviations

AAb: Autoantibodies
AC: Anti-cell
AD: Autoimmune diseases
AMA: Antimitochondrial antibodies
ANA: Antinuclear antibody
BMI: Body mass index
CFPiP: College of Family Physician in Poland
CVDs: Cardiovascular diseases
DFS70: Dense fine speckled
Hep-2: Human laryngeal carcinoma cell line
ICAP: International Consensus on ANA patterns
IIFA: Indirect immunofluorescence assay
NAAbs: Natural autoantibodies
NuMA: Nuclear mitotic apparatus
PoLA: Polish Lipid Association
SARDs: Systemic autoimmune rheumatic diseases
WHR: Waist–hip ratio
